# Low-Hysteresis and Fast Response Time Humidity Sensors Using Suspended Functionalized Carbon Nanotubes

**DOI:** 10.3390/s19030680

**Published:** 2019-02-07

**Authors:** Shivaram Arunachalam, Ricardo Izquierdo, Frederic Nabki

**Affiliations:** Department of Electrical Engineering, École de Technologie Supérieure, Montreal, QC H3C 1K3, Canada; ricardo.izquierdo@etsmtl.ca (R.I.); Frederic.Nabki@etsmtl.ca (F.N.)

**Keywords:** carbon nanotubes, suspended beam, non-suspended beam, humidity sensing, functionalized

## Abstract

A humidity sensor using suspended carbon nanotubes (CNTs) was fabricated using a low-temperature surface micromachining process. The CNTs were functionalized with carboxylic acid groups that facilitated the interaction of water vapor with the CNTs. The humidity sensor showed a response time of 12 s and a recovery time of 47 s, along with superior hysteresis and stable performance. The hysteresis curve area of the suspended structure is 3.6, a 3.2-fold reduction in comparison to the non-suspended structure. A comparative study between suspended and non-suspended devices highlights the advantages of using a suspended architecture.

## 1. Introduction

Carbon nanotubes (CNTs), first discovered by Iijima [1], are one of the most studied materials for gas sensing applications [2]. The inherent properties of CNTs, such as high surface area to volume ratio and a hollow structure, along with the ease of surface modification, have led to the use of both single- and multiwalled CNTs to detect a variety of gases with much success [3]. Sensing mechanisms can include change in resistance [4], capacitance [5,6], frequency [7], etc. To date, most of the humidity sensors based on CNTs have been based on functionalized CNTs and have shown considerable promise for commercial usage. Humidity sensors have considerable usage in household, automotive, and medical applications [8]. The performance and stability of sensors are important parameters that are the subject of continuous research using different materials and methods [9]. Fast response and recovery times and low hysteresis of a humidity sensor are important parameters to consider, and recovery performance during prolonged exposure to high humidity levels is of interest for sensors to be commercially deployed. Accordingly, these parameters are considered in this work for the proposed devices. 

There are many works in the literature focusing on the fabrication of humidity sensors based on CNTs. Multiwalled CNTs (MWCNTs)-based humidity sensors were fabricated and studied by many research groups (e.g., [5,10,11]). An ultrafast humidity sensor was fabricated by Chan et al. [12] using composite films of polyimide and MWCNTs with a response time of 5 s and a recovery time of 8 min. Liu et al. [13] developed a MWCNTs-based humidity sensor deposited by dielectrophoresis on interdigitated electrodes. Yeow et al. [14] used MWCNTs deposited on a stainless-steel substrate to fabricate a capacitive humidity sensor with a response time of 75 s. An MWCNTs-based humidity sensor was studied using different testing frequencies (AC current) with a response time of 16 s [15]. Researchers have also used single-walled CNTs to detect humidity [16]. However, most of the works mentioned above focus on the hysteresis performance of the proposed sensors. Accordingly, this work aims at characterizing this important parameter as well. 

Suspended CNTs have also been used previously to detect gases such as NO_2_ [17]. One of the main advantages of using a suspended architecture is the significantly reduced hysteresis in the devices [18]. This, combined with the increased surface area of adsorption is ideal for sensing applications. This has been demonstrated in our previous work [19]. Reduced hysteresis in a sensor leads to increased reliability and measurement consistency, which are two important parameters for sensor performance. However, suspended CNTs are difficult to fabricate and require complex and often expensive tools. There is also the question of yield in traditionally followed methods to obtain suspended CNTs where individual CNTs or a cluster of CNTs are grown between pre-fabricated electrodes using high-temperature chemical or physical vapor deposition processes. This approach is generally used to study intrinsic properties such as transport phenomena [20], phonon interactions [21], etc. Thus, suspended CNTs have rarely been used in sensing applications, even though they offer significant inherent advantages. 

In our previous work [19], the advantages of pristine suspended CNTs have been demonstrated. The suspended CNTs showed near-zero hysteresis and better response and recovery times than non-suspended CNTs. Moreover, the fabrication process proposed had a low temperature budget, making the sensors suitable for integration above integrated circuits. However, the response and recovery times were relatively long, curtailing commercial viability. This work is an improvement over the previous work by using functionalized single-walled CNTs. The suspended functionalized CNTs also showed near zero hysteresis, remarkable repeatability along with fast response and recovery times. To further demonstrate the advantages of using a suspended architecture, the device performance is compared to a traditional non-suspended CNT device using the same fabrication process.

## 2. Materials and Methods

The detailed fabrication process has been discussed previously in [19]. Notably, the fabrication process features a very low temperature budget (i.e., below 110 °C) and is amenable to integration above integrated circuits. The following is a brief overview of the fabrication process. 

**Figure 1:**
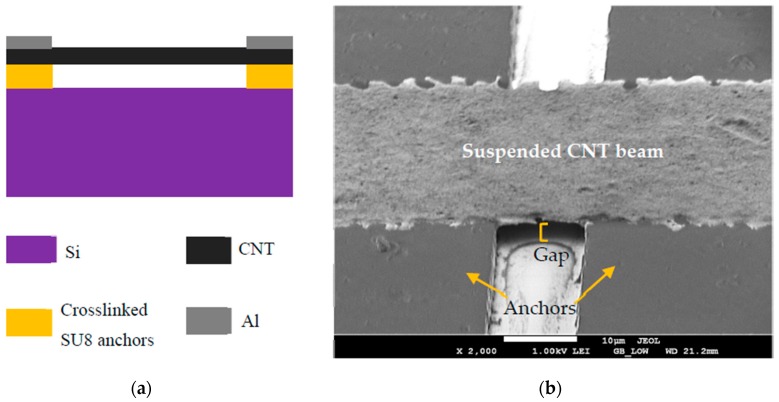
(**a**) Schematic of the suspended CNT beam (**b**) SEM micrograph.

The process is carried out on a silicon (Si) substrate. SU8, an epoxy-based negative photoresist is used as a sacrificial layer to obtain suspended CNTs. SU8 was chosen as the sacrificial material due to its uniform surface profile and ease of availability. The SU8 is partially crosslinked by UV lithography. The crosslinked SU8, of thickness 3.6 µm, is used as an anchor for the CNT beam, while the uncrosslinked SU8 is used as a sacrificial layer. The uncrosslinked SU8 is prevented from dissolving in further processing steps by depositing a 60-nm barrier layer of aluminum by filament evaporation. The CNTs used in this work are obtained pre-functionalized with a carboxylic acid group (‒COOH) from Carbon Solutions Inc. (Riverside, CA, USA) and used without any further material processing. 0.125 g of the as-obtained CNTs in powder form were dispersed in a 1 wt % SDS solution. The as-prepared solution was then ultrasonicated for 6 h at room temperature, resulting in a uniformly dispersed CNT solution. The solution is then ultra-centrifuged at 47,300 rpm for 60 min. The top half of the solution is then decanted for further use. Then, the CNT films are formed using vacuum filtration. Vacuum filtration enables the formation of a homogenous film with a uniform distribution of CNTs. This process also ensures strict control over film thickness. The CNT films used here are 0.7 µm thick. The 20 nm thick aluminum (Al) electrodes are deposited on the CNTs before release to form the suspended CNT beams. The schematic of the resulting suspended CNT beam cross section is shown in Figure 1a and a SEM micrograph of a fabricated beam is shown in Figure 1b. 

## 3. Results

The device characterization is done in a humidity chamber connected to an external humidifier, which acts as the humidity source. The sensors are interconnected using probes aligned using micro-positioners. The humidity percentage in the chamber was verified using a high-precision sensor embedded within the chamber. The resistance of the devices was measured using a Keithley Digital Multimeter (Cleveland, OH, USA) connected to a computer for data acquisition. 

### 3.1. Humidity Response

**Figure 2:**
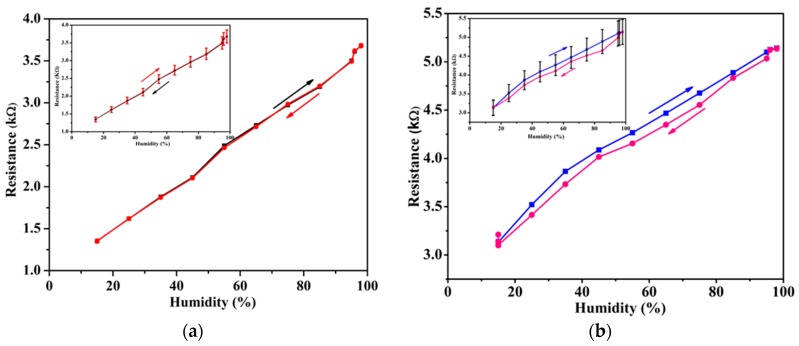
(**a**) Plot of the humidity response of the suspended CNTs, and (**b**) Plot of the humidity response of the non-suspended CNTs. The hysteresis characteristic is also shown. The insets in each figure show the average hysteresis profile of 6 identical devices measured under similar testing conditions.

To study the humidity sensing properties of the CNT-based sensors, the resistance of the devices was measured as a function of relative humidity. The humidity was measured from a base of 15% RH to a maximum of 98% RH gradually increased in steps of ∼10% RH per 2 min. The results are shown in Figure 2 for both suspended and non-suspended devices. The inset plots of Figure 2a,b show the average hysteresis profile of six measured devices of each type (i.e., suspended and non-suspended) while the outset shows the device response of a typical device of each type. A comparison of the hysteresis profile of a single device to the average profile shows that the suspended devices have a consistent response to humidity. The average base resistance of the suspended CNTs for six measured devices at 15% RH is 1.3 kΩ and it rises to an average of around 3.5 kΩ at 98% RH. In comparison, the non-suspended devices had an average base resistance of 3.3 kΩ at 15% RH and 5.1 kΩ at 98% RH. Moreover, the suspended devices exhibit minimal hysteresis with an average hysteresis curve area of 3.56, even after eight measurement cycles. In comparison, the non-suspended sensors exhibited significant hysteresis, with an average hysteresis curve area of 11.21. This represents a 3.2-fold improvement in the hysteresis behavior of the suspended sensor over the non-suspended sensor. The hysteresis curve area was calculated using the in-built Integrate function of the Origin software package. The lack of hysteresis in the suspended sensor can be attributed to the lack of substrate effects such as charge traps [18], which could alter the pathway of the charge carriers in the nanotube network. The charge traps and grain boundaries within the substrate alter the conductivity and hence the non-suspended sensor also shows higher resistance. 

### 3.2. Response Time, Recovery Time, and Sensitivity

**Figure 3:**
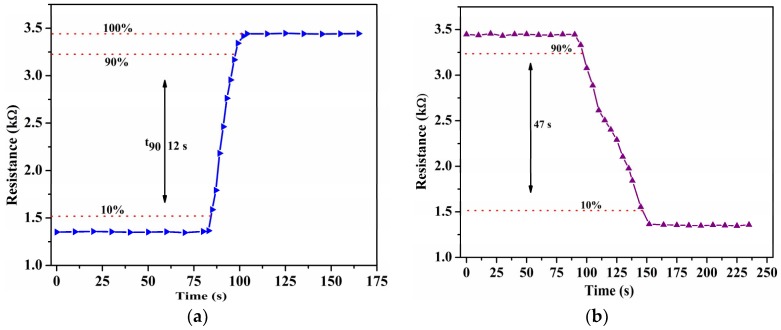

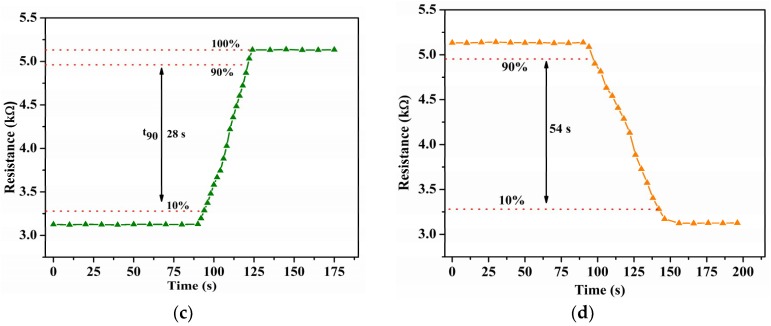
(**a**) Response time and (**b**) recovery time of the suspended CNTs; (**c**) response time and (**d**) recovery time of the non-suspended CNTs. The humidity was increased gradually in steps of 10% RH.

The response time, defined as the time taken to achieve 10‒90% of the total resistance change, is measured to be 12 s for the suspended sensors and around 28 s for the non-suspended sensors, as shown in Figure 3a,c, respectively. The 2.3-fold shorter response time of the suspended sensors is due to the increased surface area for the adsorption of water molecules. Since the film is suspended, the molecules can adhere to both the top and bottom surface of the CNT beam. The recovery time, defined as the time taken by the sensor to go from 90% to 10% of its original resistance value is measured to be 47 s for the suspended sensors and 54 s for the non-suspended sensors, as shown in Figure 3b,d, respectively. 

**Figure 4:**
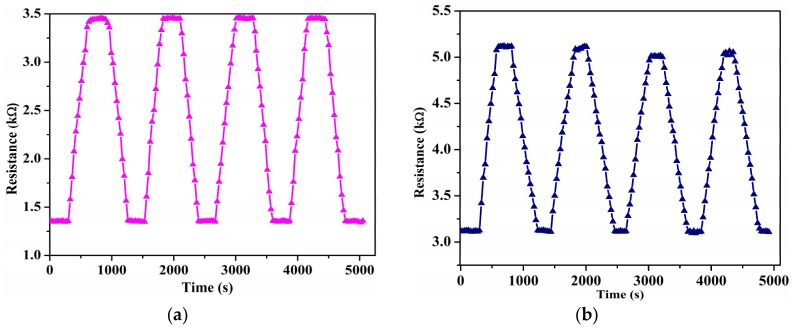
(**a**) Repeatability measurement over four humidity cycles for the suspended CNTs, and (**b**) for the non-suspended CNTs.

Consistent performance of the sensor is necessary for long-term applications. Thus, repeatability of the sensors was verified by subjecting them to continuous humidity cycles by varying humidity from 15% to 98% RH and recording the resistance at an interval of every 5 s. As seen in Figure 4a, the suspended sensors showed remarkable stability and repeatability. In comparison, the non-suspended sensors showed relatively stable performance with a drift in performance after three humidity cycles, as shown in Figure 4b. This further demonstrates the advantages of using suspended CNTs over traditional non-suspended CNTs. The excellent repeatability is due to the ease of adsorption and desorption of the water molecules from the surface of the suspended CNT beam. 

**Figure 5:**
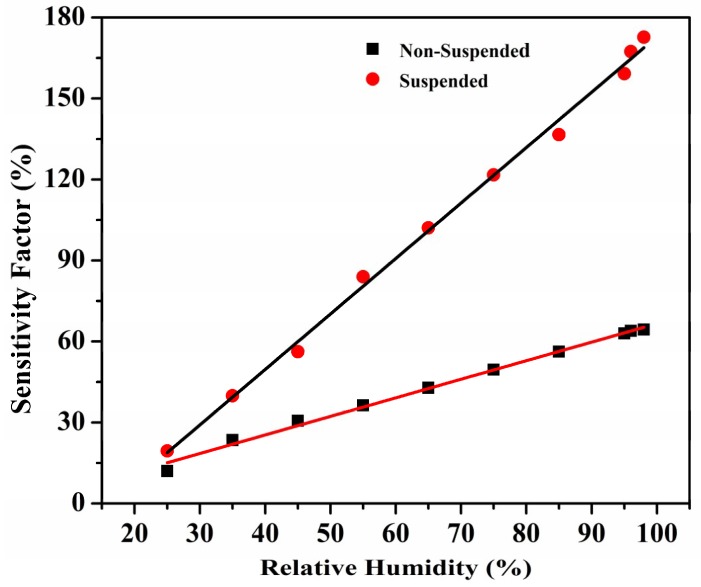
Sensitivity factors of suspended CNTs vs. non-suspended CNTs.

The humidity sensing mechanism of CNTs has been studied and discussed extensively in previous works [10]. The sensing is primarily due to charge transfer within the CNT networks. The CNTs are inherently p-type. As the water molecules donate electrons to the CNTs as a result, the number of primary charge carriers in the CNTs (holes) decreases and thus the resistance increases in the sensor. The sensitivity factor is given by [10,22]:

(1) $$S=\frac{R_{H}-R_{0}}{R_{0}}\times 100\%,$$

where *R_H_* is the resistance at any given humidity, *R*_0_ is the starting resistance at 15% RH. The sensitivity factor comparison of the suspended and non-suspended sensors is shown in Figure 5. At 98% humidity, the sensitivity factor of the suspended sensor is 172.9% compared to 64.2% for the non-suspended sensor. This outlines a 2.7-fold improvement for the suspended structure. The slope of the graph, which represents the percentage change in the resistance from the initial resistance per % RH change, for the suspended sensor is 2.05 compared to 0.68 for the non-suspended sensor, meaning the sensitivity of the suspended sensor is 3-fold greater than the non-suspended sensor. Demonstrably, the suspended sensor shows better performance in all aspects and thus is a better prospect for CNT-based humidity sensors. The acidic functionalization of CNTs shortens the CNTs, making ends open and introduces side wall defects, which facilitate water adsorption. The increased water adsorption capacity combined with shortened pathways for the charge carriers result in faster response times in the functionalized CNT sensor [23].

### 3.3. Temperature Study

**Figure 6:**
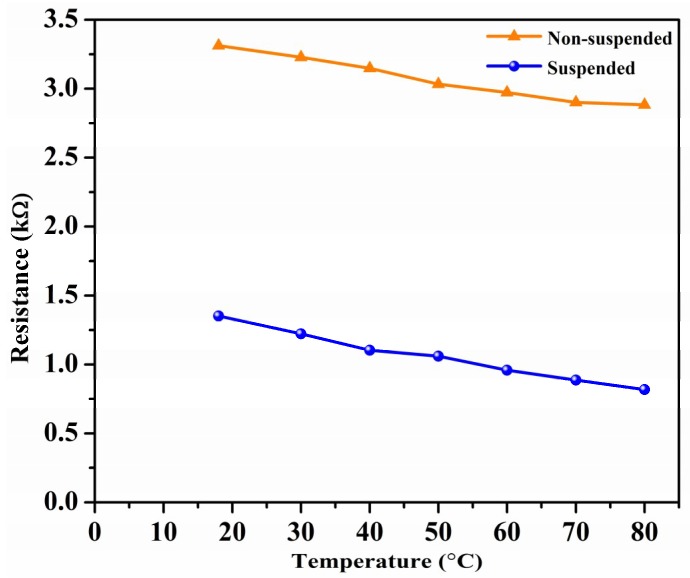
Resistance vs. temperature for suspended and non-suspended CNTs.

The resistance of the suspended devices was measured at different temperatures to gauge the performance under different thermal operating conditions. In Figure 6, the resistance of the suspended and non-suspended sensors as a function of temperature is plotted. As can be seen, the resistance of the devices decreases with an increase in temperature. This is attributed to a decrease in the number of water molecules available to interact with the CNT networks at higher temperatures. Moreover, the thermal energy of the electrons provided by the water molecules increases with increasing temperature. The imparted thermal energy is enough for the electrons to overcome the potential barrier between the CNTs in the network, which results in a decrease in the film’s resistance [24,25]. 

### 3.4. Long-Term Stability

**Figure 7:**
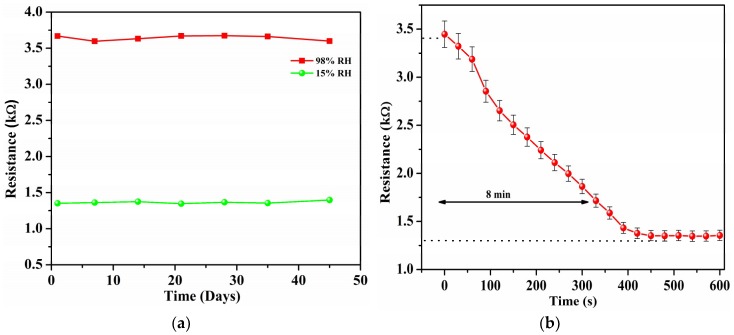
(**a**) Resistance as a function of time for suspended CNTs, indicating device stability, and (**b**) recovery time to 15% RH of the suspended CNTs after exposure to 98% RH for 24 h.

The long-term stability of a sensor is a major factor in determining its performance. To gauge long-term stability, the resistance of the suspended device was measured at regular intervals of seven to 10 days. Figure 7a shows the resistance value of the sensor at two different humidity levels over a 45-day period. This demonstrates the stability of the sensor over extended periods as the resistance fluctuates less than 3.32% over the timespan of the test. To gauge the sensor recovery time after prolonged exposure, the sensor was soaked in a humid environment at 98% RH for 24 h.

The humidity was then flushed out and the sensor response was recorded. Figure 7b shows the graph of the sensor recovery after prolonged exposure. The sensor took approximately 8 min to recover from 90% to 10% of its original resistance. In addition, no permanent shift in the sensor resistance was exhibited after recovery. Table 1 shows a comparison of the response and recovery times of few of the different CNT humidity sensors in literature. The presented sensor exhibits good response and recovery times compared to other works. In addition, the device also exhibits enhanced sensitivity factor when compared to the other works and a 3-fold improved sensitivity than a non-suspended sensor. 

## 4. Conclusions

In this work, a humidity sensor based on a suspended carbon nanotube has been demonstrated. The suspended sensor showed good performance with near-zero hysteresis, fast response and recovery times, and exhibited good long-term stability. To demonstrate the advantages of using a suspended architecture, the performance of suspended CNTs was compared to a non-suspended sensor under similar testing conditions. Evidently, the suspended sensors showed superior humidity sensing characteristics such as near-zero hysteresis, and significantly improved sensitivity owing to lack of substrate effects and more surface area for adsorption. Moreover, the work improved the response time of the devices as compared to our previous work in [19] by more than 10-fold due to the chemical functionalization of the nanotubes making them more sensitive to the water molecules. The suspended humidity CNT sensor presented here also compares favorably in terms of response and recovery times to other literary works. The proposed sensor design thus has the potential of yielding high-performance humidity sensors that are suitable to integration with integrated circuits. 

## Figures and Tables

**Table 1:** Comparison of the response and recovery times of different CNT humidity sensors.

Publication	Sensing Material	Response Time (s)	Recovery Time (s)	Sensitivity Factor (%)
This work	Functionalized Single-walled CNTs	12	47	172.9
Jung et al. [22]	Metal oxide coated CNTs	30	25	~60
Cao et al. [10]	Functionalized Multiwalled CNTs	50	140	124
Mudimela et al. [16]	Single-walled CNTs	180	240	N/A
Zhang et al. [26]	Multiwalled CNTs	60	70	80
Moraes et al. [27]	Functionalized Multiwalled CNTs	3	90	135
Chen et al. [5]	Multiwalled CNTs	16	8	29.9
Arunachalam et al. [19]	Single-walled CNTs	290	510	246.9
